# INI-1 Loss with brachyury positivity unmasking a cervical poorly differentiated chordoma initially diagnosed as epithelioid sarcoma: a case report

**DOI:** 10.3389/fsurg.2026.1807700

**Published:** 2026-05-22

**Authors:** Murat Baloglu, Mehmet Emin Akdeniz, Kivanc Yangi, Celal Ozbek Cakir, Nazli Sena Seker, Ismail Bozkurt

**Affiliations:** 1 Dept. of Neurosurgery, Eskisehir City Hospital, Eskisehir, Turkiye; 2 Dept. of Neurosurgery, Prof. Dr. Cemil Tascioglu City Hospital, Istanbul, Turkiye; 3 Dept. of Neurosurgery, Barrow Neurological Institute, St. Joseph’s Hospital and Medical Center, Phoenix, AZ, United States; 4 Dept. of Neurosurgery, Afyon Park Hospital, Afyon, Turkiye; 5 Dept. of Pathology, Faculty of Medicine, Eskişehir Osmangazi University, Eskişehir, Turkiye; 6 Dept. of Neurosurgery, Medical Park Ankara Hastanesi, Yüksek İhtisas Üniversitesi Tıp Fakültesi, Ankara, Turkiye

**Keywords:** brachyury, cervical spine, chordoma, epithelioid sarcoma, poorly-differentiated chordoma

## Abstract

**Introduction:**

Poorly-differentiated chordoma is a rare, aggressive tumor that can mimic epithelioid sarcoma, particularly when biopsies show epithelioid morphology with INI-1 loss.

**Case presentation:**

A 31-year-old man presented with neck pain and a cervical mass and underwent resection in 2018, followed by radiotherapy, chemotherapy, and spinal stabilization. The lesion was initially classified as epithelioid sarcoma, and subsequent recurrences in 2022 and 2025 were interpreted similarly. However, re-evaluation of all specimens demonstrated pan-keratin positivity, loss of INI-1, and nuclear brachyury expression, prompting revision of the diagnosis to poorly differentiated chordoma.

**Results & conclusion:**

This case underscores the importance of early brachyury testing in INI-1–deficient axial or paraspinal epithelioid tumors to avoid misdiagnosis and guide appropriate management.

## Introduction

Poorly differentiated chordoma (PDC) is a rare, aggressive subtype of chordoma characterized by SMARCB1/INI-1 loss, predominantly affecting children and young adults, and posing significant diagnostic challenges, particularly when biopsy material is limited (1–5). It predominantly involves the axial skeleton, with a particular predilection for the skull base and cervical spine, whereas chordomas, overall, most commonly arise in the skull base and sacrococcygeal region (6, 7). In particular, an axial/paraspinal tumor with epithelioid morphology and SMARCB1/INI-1 loss may be misclassified as epithelioid sarcoma because of morphologic and immunophenotypic overlap among INI-1–deficient epithelioid neoplasms, leading to major downstream consequences for treatment planning and surveillance (3, 8). In this setting, early use of lineage-defining markers is essential: nuclear brachyury (TBXT) is highly specific for chordoma and can reliably resolve this critical differential diagnosis when morphology and INI-1 status are misleading (9). Here, we report a 31-year-old man who presented with left-sided neck pain and a gradually enlarging left cervical mass beginning in late 2017, underwent multiple surgical procedures between 2018 and 2025, and was treated as epithelioid sarcoma until late 2025; however, the diagnosis was ultimately revised to poorly differentiated chordoma after extended immunohistochemistry demonstrated nuclear brachyury positivity, with follow-up ongoing in 2026.

## Case report

### Clinical course and management

A 31-year-old man was evaluated for several months of progressive left-sided neck pain and a gradually enlarging left cervical mass, at the level of C2-C3 vertebrae, that had started in late 2017. On presentation and throughout the documented course, neurological examinations were reported as noncontributory, with no new postoperative deficits after subsequent interventions. At the time of his first admission in early 2018, he reported no comorbidities, no regular medications, and no prior surgeries; no family history or psychosocial factors relevant to the presenting complaint were noted in the available records.

**Figure 1:**
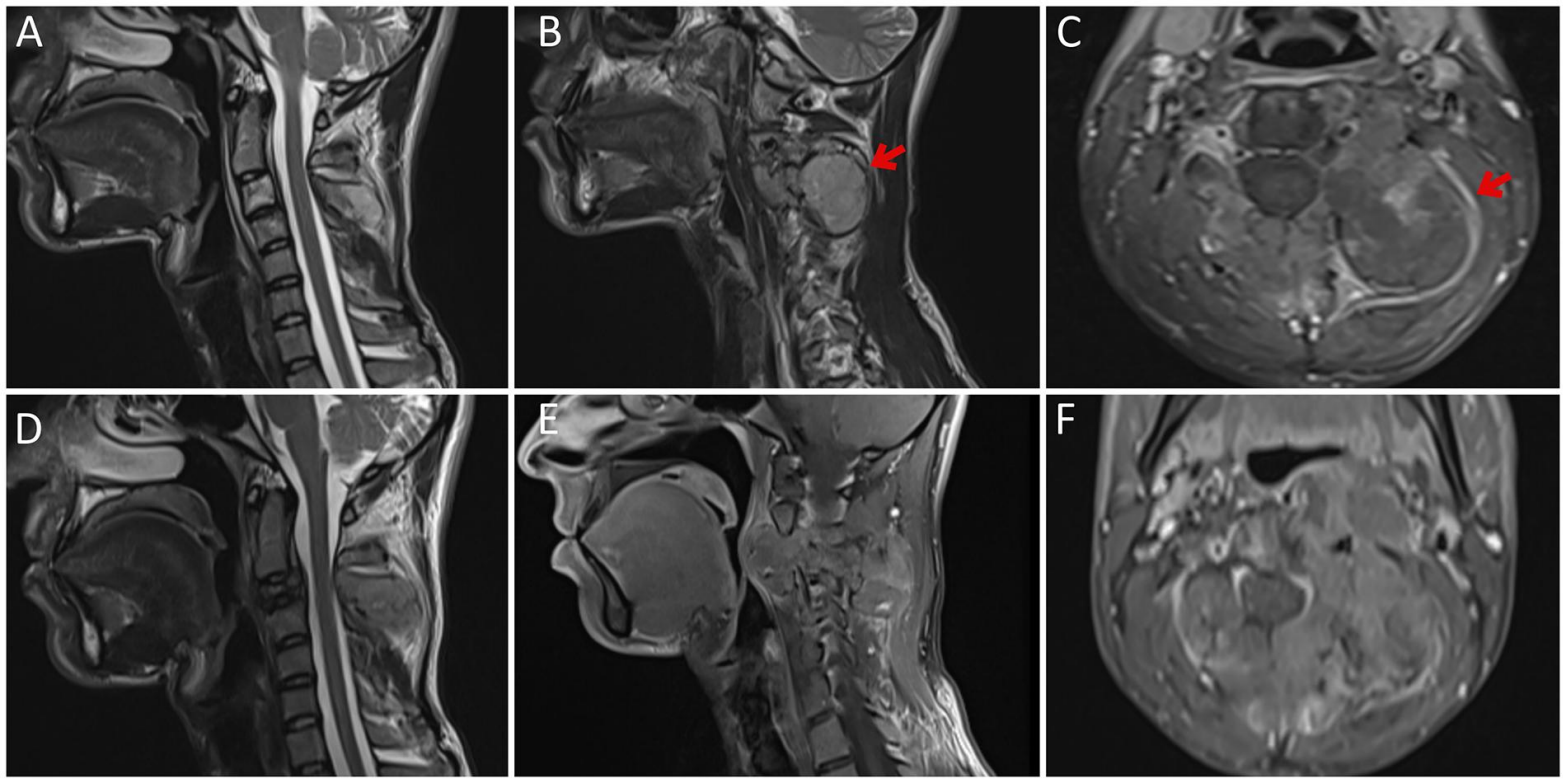
Cervical magnetic resonance imaging (MRI) in March and April 2018. In March 2018, **(A)** a sagittal T2-weighted image demonstrates preserved cervical cord caliber and signal without an intraspinal mass; **(B)** an off-midline sagittal T2-weighted image shows a left posterior paraspinal upper cervical mass (red arrow); and **(C)** an axial post-contrast T1-weighted image demonstrates peripheral enhancement of the lesion extending toward the left C3 neural foramen (red arrow). In April 2018, **(D)** a midline sagittal T2-weighted image demonstrates interval progression with vertebral body collapse at the C3 level; **(E)** an off-midline sagittal post-contrast T1-weighted image shows expansion of the paraspinal mass with anterior extension toward the retropharyngeal region; and **(F)** an axial post-contrast T1-weighted image demonstrates mass effect with narrowing of the pharyngeal airway.

Cervical magnetic resonance imaging (MRI) in March 2018 demonstrated a left posterior paraspinal upper cervical mass extending toward the neural foramina of C2-C3, approximately 47 × 38 × 45 mm, with associated osseous destruction and peripheral enhancement on post-contrast images. Its craniocaudal extent ranged from the upper margin of the C2 vertebral body to the lower margin of the C4 vertebral body without an intraspinal component, and the cervical cord caliber and signal were preserved (Figure 1). One month later (April 2018), a repeat contrast-enhanced MRI showed interval progression with collapse of the C3 vertebral body and expansion of the paraspinal mass with anterior extension toward the retropharyngeal region and narrowing of the oropharyngeal airway (Figure 1), corresponding to newly reported dysphagia, and reported possible encasement and compression of the adjacent vertebral artery. In May 2018, the patient underwent surgical resection for the cervical lesion, and the operative pathology was reported as a malignant mesenchymal tumor interpreted as epithelioid sarcoma. Following surgery, the patient received adjuvant radiotherapy to the involved cervical region (total 50 Gy, documented as 28 fractions) in June 2018, followed by six cycles of doxorubicin–ifosfamide chemotherapy completed in December 2018, and was then maintained on pazopanib with radiological surveillance. Pazopanib was initiated by the treating medical oncologist as maintenance therapy following completion of adjuvant chemotherapy. Although pazopanib is approved for metastatic non-adipocytic soft-tissue sarcoma after previous chemotherapy based on the PALETTE trial (10), its use in this setting represented an off-label but clinically reasoned extension of the available evidence under the working diagnosis of epithelioid sarcoma.

**Figure 2:**
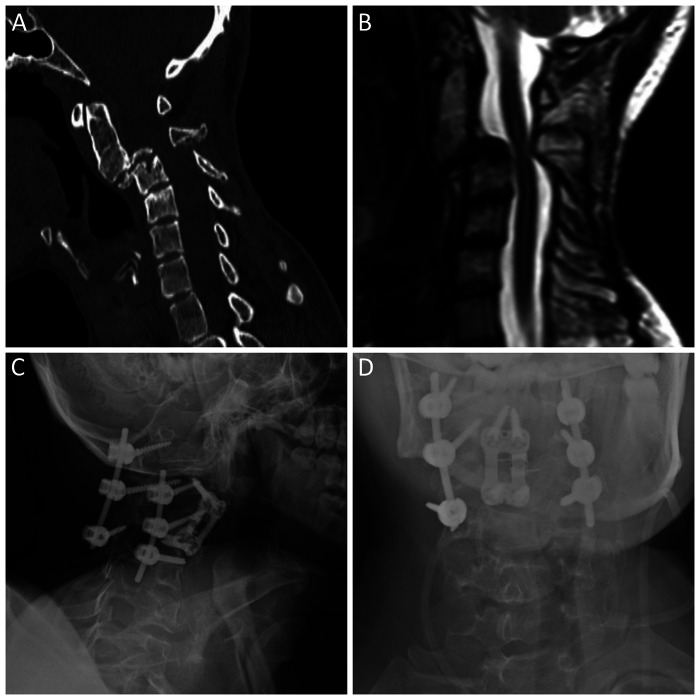
Preoperative imaging and postoperative radiographs demonstrating severe cervical instability and staged 360-degree stabilization. In December 2020, a preoperative sagittal computed tomography (CT) scan **(A)** demonstrated severe C2–C3 anterolisthesis with marked C3 vertebral body collapse, and **(B)** a sagittal T2-weighted MRI demonstrated marked spinal canal narrowing at the C2–C3 level. Postoperative radiographs demonstrate **(C)** a lateral view showing anterior C3 corpectomy with C2–C4 cage reconstruction and anterior plate fixation combined with posterior screw–rod instrumentation spanning C1–C4, and **(D)** an anteroposterior view confirming the combined anterior C2–C4 construct and posterior instrumentation.

In December 2020, during follow-up, the patient was found to have severe mechanical instability with grade 4 C2–C3 anterolisthesis and marked spinal canal narrowing (anteroposterior diameter approximately 8.3 mm), with the C3 vertebral body showing approximately 90% height loss (Figures 2A,B). The patient subsequently underwent staged 360-degree cervical stabilization, consisting of anterior C3 corpectomy with C2–C4 cage reconstruction and anterior plate fixation, followed by posterior screw–rod instrumentation spanning C1 to C4 with C2 laminectomy (Figures 2C,D). He recovered without a new neurological deficit and continued oncologic follow-up. In 2022, follow-up cervical MRI was substantially limited by susceptibility artifact from prior instrumentation, representing a key diagnostic challenge, and residual disease could not be reliably assessed. No definite mass lesion was identified, and imaging was reported as stable compared with the prior study. Around the same period, a repeat biopsy at an outside center was documented as a malignant mesenchymal tumor, and the recorded diagnosis remained unchanged. Follow-up imaging in December 2024 showed no definite residual or recurrent mass. However, in June 2025, surveillance imaging demonstrated a recurrent tumor adjacent to the left-sided cervical instrumentation levels, extending toward the spinal canal with dural indentation.

**Figure 3:**
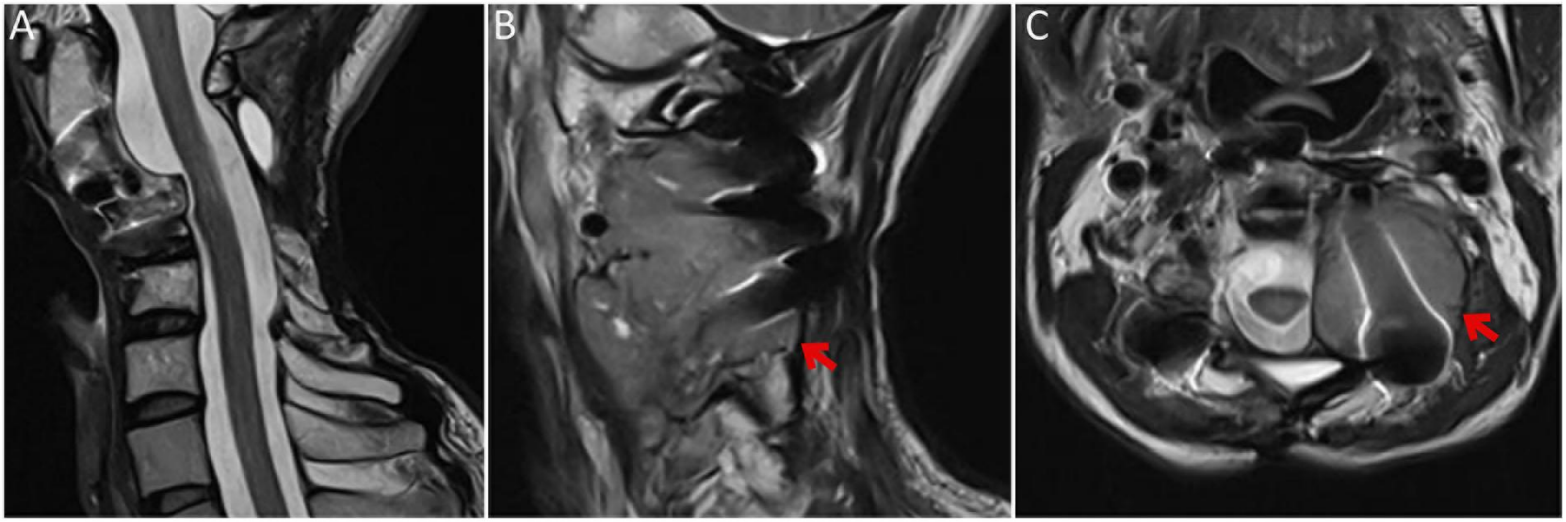
Preoperative cervical MRI in November 2025. T2-weighted images show **(A)** preserved cervical cord caliber and signal on a midline sagittal view, **(B)** an off-midline sagittal view demonstrating a recurrent left cervical paraspinal mass, and **(C)** an axial T2-weighted image showing dural indentation despite susceptibility artifact from prior instrumentation. Red arrows indicate the tumor in **(B,C)**.

In August 2025, a fine-needle biopsy from the recurrent lesion was interpreted as malignant tumoral infiltration with epithelioid morphology and loss of nuclear INI-1 expression, supporting recurrence under the prior epithelioid sarcoma diagnosis. In November 2025, due to persistent left-sided neck pain and radiological progression (Figure 3), the patient underwent excision of recurrent tumor tissue from the left cervical paraspinal region. Neurological examinations before and after surgery remained unremarkable. The postoperative course was reportedly uneventful, and the patient was discharged on postoperative day 2. The postoperative specimen was initially reported as malignant tumoral infiltration, and additional immunohistochemistry was requested. A consultation review at the referral center re-evaluated archived specimens from 2018, 2022, and 2025. Across multiple time points, tumor cells showed pan-keratin expression with loss of nuclear INI-1 and, importantly, nuclear brachyury positivity, leading to a revised diagnosis of poorly differentiated chordoma.

At the last documented follow-up after the November 2025 excision, the patient remained neurologically intact, and ongoing radiologic surveillance was planned. The clinical timeline is summarized in Table 1.

**Table 1:** Chronological summary of clinical events, imaging findings, pathological evaluations, and treatments.

Date	Event
Late 2017	Onset of progressive left-sided neck pain and a gradually enlarging left cervical mass
March 2018	Cervical MRI: left posterior paraspinal upper cervical mass (47 × 38 × 45 mm) with osseous destruction and peripheral enhancement
April 2018	Repeat MRI: interval progression with C3 vertebral body collapse, retropharyngeal extension, and oropharyngeal airway narrowing
May 2018	Surgical resection of cervical lesion; pathology reported as epithelioid sarcoma
June 2018	Adjuvant radiotherapy (50 Gy in 28 fractions) to the involved cervical region
June–December 2018	Six cycles of adjuvant doxorubicin–ifosfamide chemotherapy
December 2018	Pazopanib maintenance therapy initiated following completion of adjuvant chemotherapy, under the working diagnosis of epithelioid sarcoma
December 2020	Imaging: grade 4 C2–C3 anterolisthesis with marked spinal canal narrowing; C3 vertebral body approximately 90% height loss. Staged 360-degree cervical stabilization performed (anterior C3 corpectomy with C2–C4 cage reconstruction and posterior C1–C4 screw–rod instrumentation)
2022	Follow-up MRI limited by instrumentation artifact; no definite mass identified. Outside biopsy at a separate center: diagnosis of epithelioid sarcoma maintained
December 2024	Follow-up imaging: no definite residual or recurrent mass
June 2025	Surveillance MRI: recurrent tumor adjacent to left cervical instrumentation with extension toward the spinal canal and dural indentation
August 2025	Fine-needle biopsy: malignant tumoral infiltration with epithelioid morphology and INI-1 loss, supporting recurrence under the prior epithelioid sarcoma diagnosis
November 2025	Surgical excision of recurrent tumor. Consultation review of archived specimens (2018, 2022, and 2025) demonstrated pan-keratin positivity, INI-1 loss, and nuclear brachyury positivity; diagnosis revised to poorly differentiated chordoma
Last follow-up	Patient neurologically intact; ongoing radiologic surveillance planned

### Histopathological analysis

**Figure 4:**
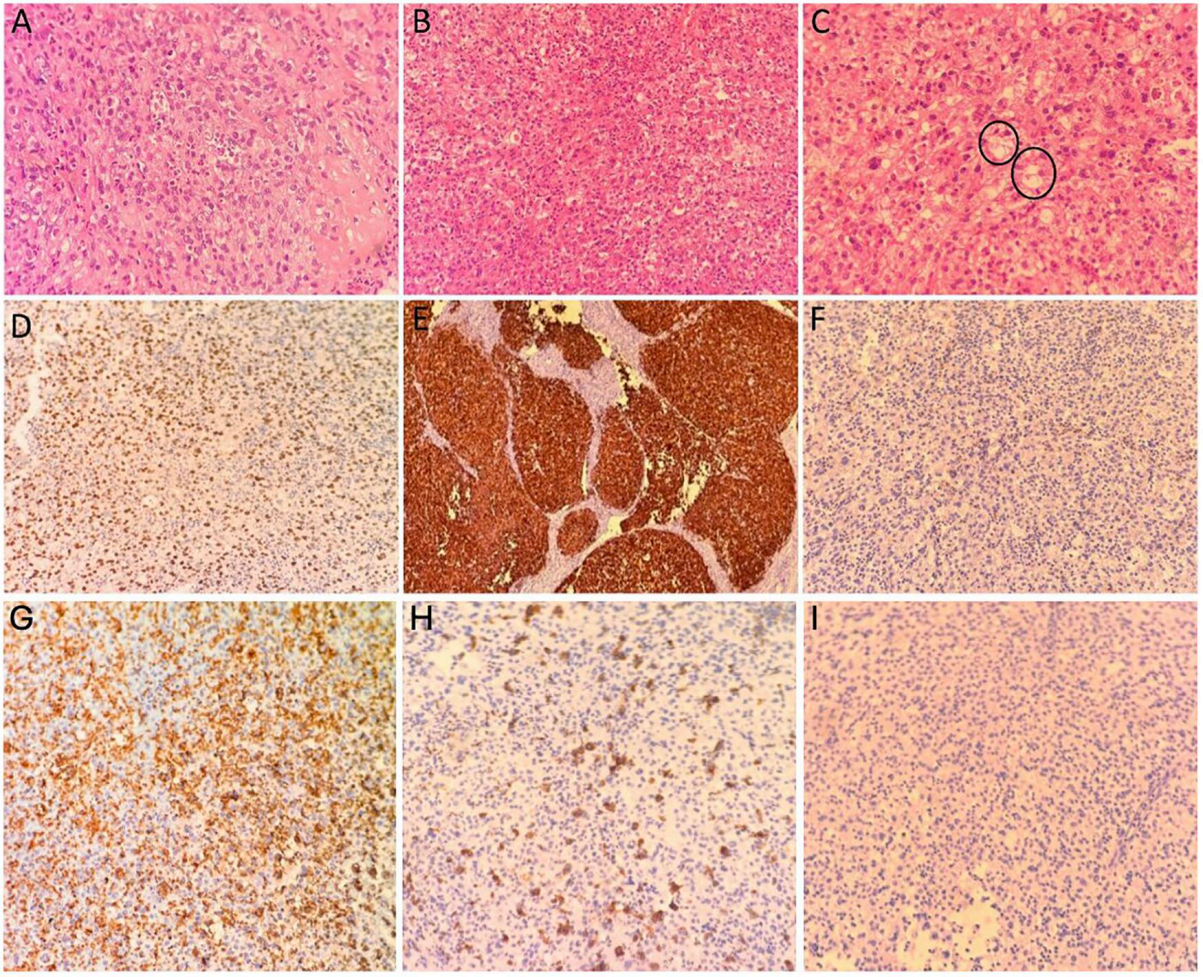
Histopathological and immunohistochemical findings. H&E-stained sections showed **(A)** malignant tumoral infiltration with epithelioid morphology (×200), **(B)** numerous neutrophilic leukocytes accompanying the tumor cells (×200), and **(C)** tumor cells with vacuolated cytoplasm (circled) (×200). Immunohistochemistry demonstrated **(D)** nuclear brachyury positivity (×200), **(E)** diffuse pan-keratin positivity (×40), and **(F)** loss of nuclear INI-1 (SMARCB1) expression in tumor cells (×100), supporting the diagnosis of poorly differentiated chordoma **(G)** Numerous neutrophilic leukocytes surrounding the tumor cells (MPO IHC, ×200) **(H)** Scattered histiocytes surrounding the tumor cells (CD68 IHC, ×200) **(I)** Absence of T lymphocytes in the tumor microenvironment (CD3 IHC, ×200).

The hematoxylin–eosin–stained sections revealed a neoplastic cellular infiltration composed of epithelioid cells arranged predominantly in a nodular growth pattern with prominent eosinophilic nucleoli. Marked pleomorphism and nuclear atypia were present, with focal areas of necrosis. Scattered tumor cells with vacuolated cytoplasm were also identified, along with a mixed inflammatory infiltrate (Figure 4). Given the epithelioid morphology, axial/paraspinal location, and dispersed vacuolated cells, the differential diagnosis included metastatic carcinoma, epithelioid malignant peripheral nerve sheath tumor, alveolar soft part sarcoma, epithelioid sarcoma, and poorly differentiated chordoma. The initial surgical specimen was evaluated at an external pathology laboratory in 2018 using an immunohistochemical panel including pan-cytokeratin, CD34, INI1, S100, SOX10, TFE3, and ERG. Following referral to our institution for consultation, additional brachyury immunostaining was performed.

Immunohistochemistry demonstrated nuclear positivity for brachyury and pan-keratin, accompanied by loss of nuclear INI-1 (SMARCB1) (Figure 4). Tumor cells were negative for CD34, S100, SOX10, TFE3, and ERG. Numerous MPO-positive neutrophilic leukocytes and scattered CD68-positive histiocytes were observed surrounding the tumor cells. No staining was detected with the applied lymphoid marker (Figure 4). Based on the combined morphologic and immunophenotypic findings, particularly nuclear brachyury positivity, the final diagnosis was established as poorly differentiated chordoma.

### Patient informed consent

Written informed consent was obtained from the patient for participation and publication. This case report was prepared in accordance with the CARE reporting guidelines (11).

## Discussion

This case highlights a clinically important diagnostic pitfall in axial/paraspinal epithelioid neoplasms: a cervical paraspinal tumor managed for years as epithelioid sarcoma was ultimately reclassified as poorly differentiated chordoma after consultation review demonstrated nuclear brachyury (TBXT) positivity together with loss of INI-1 (SMARCB1). Beyond refining lineage assignment, this reclassification has practical implications for treatment strategy and follow-up. Poorly differentiated chordoma is characterized by loss of INI-1 expression, creating a diagnostic pitfall with epithelioid sarcoma, particularly on limited biopsy specimens or when brachyury is not included in the initial immunohistochemical panel (2, 8, 9, 12). Large series and molecular studies emphasize that poorly differentiated chordoma often presents diagnostic challenges because morphology and a “generic” epithelial profile, such as keratin expression, may not be discriminative on their own (4, 12). In this context, brachyury remains the most useful lineage-directed marker: nuclear brachyury expression strongly supports a diagnosis of chordoma and helps separate it from mimics in the appropriate anatomic setting (2, 9, 13). Therefore, in INI-1–deficient epithelioid tumors arising in the axial skeleton or paraspinal region, early inclusion of brachyury in the immunohistochemical panel is a practical step to reduce misclassification, especially when the initial differential diagnosis includes epithelioid sarcoma or a metastatic carcinoma (13). Accurate classification is clinically important because treatment paradigms diverge. For chordoma, the therapeutic backbone is maximal safe resection when feasible and high-dose radiotherapy, whereas conventional cytotoxic regimens used for soft-tissue sarcomas generally have limited and inconsistent benefit in chordoma (14, 15). In contrast, epithelioid sarcoma is typically approached within soft-tissue sarcoma frameworks and, in advanced disease, may involve targeted options such as EZH2 inhibition for INI-1–deficient tumors (16). Misclassification may therefore lead to systemic therapy choices, surveillance intensity, and counseling that do not match the biology of the underlying entity. Specifically, chordomas are considered relatively radioresistant, and international consensus recommendations favor high-dose radiation (≥74 GyE) (14, 15), whereas the 50 Gy in 28 fractions administered in the present case was consistent with a soft-tissue sarcoma adjuvant protocol (17). Similarly, doxorubicin-based chemotherapy, a cornerstone of soft-tissue sarcoma treatment (17), has limited and inconsistent efficacy in chordoma (14, 15). Recent case reports have similarly described poorly differentiated chordoma initially interpreted as epithelioid sarcoma, underscoring the practical impact of this pitfall (18). Taken together with the present case, the literature supports a straightforward diagnostic message: when an epithelioid neoplasm in an axial or paraspinal location shows INI-1 deficiency, clinicians and pathologists should include chordoma-oriented markers, particularly brachyury, in the immunohistochemical work-up to reduce misclassification and downstream mismanagement.

### SMARCB1 (INI1) loss and the immune microenvironment

SMARCB1 (INI-1) is a subunit in the SWI/SNF family involved in chromatin regulation. As is known, chromatin folds around histone proteins and forms a nucleosome. Transcription requires the dynamic opening and closing of these chromatin regions. The SWI/SNF family is the main factor responsible for this nucleosome regulation (19). In the SWI/SNF family, SMARCB1 (INI1) loss was first discovered in childhood rhabdoid tumors, establishing one of the earliest links between SWI/SNF complex dysfunction and cancer development (20). Tumor microenvironment and related treatment modalities have been increasingly investigated in recent years. However, compared with epithelial tumors, sarcomas have been less extensively studied because they represent a rare and heterogeneous group of malignancies. Notably, the study by Leruste et al. revealed that rhabdoid tumors with SMARCB1 (INI1) loss are highly immunogenic despite having a low tumor mutational burden (TMB) (21). In SMARCB1(INI1) loss tumors, it has been shown that the repression of “endogenous retroviral elements (ERVs)” is reversed, and an immune response occurs with an increase in interferon (IFN), like an antiviral response. Previous studies have also suggested that tumors with derepressed ERVs may be associated with responsiveness to anti-PD-L1 therapies (22). In tumors with SMARCB1 (INI1) loss, CD8-rich T lymphocyte infiltration is observed in the tumor microenvironment, along with CD68 + myeloid cells, dendritic cells, and neutrophils (23, 24).

In chordomas, several studies investigating the tumor microenvironment have shown a microenvironment that is generally rich in T lymphocytes and macrophages, whereas significant neutrophil infiltration has not been consistently reported (25, 26). In our case, a dense neutrophilic infiltration was observed around the tumor cells. A proteomic study integrating clinical correlations demonstrated that chordomas may exhibit location-dependent microenvironmental differences. Accordingly, neutrophil, macrophage, and stem cell regulatory signatures are observed in tumors of the spine and skull base, whereas T cell regulatory signatures are observed in those of the sacral region (27). These findings highlight the remarkable intertumoral heterogeneity of chordomas and may partly explain the neutrophil predominance observed in our case. Tumor-associated neutrophils (TANs) can exhibit different tumoral effects depending on their subtype. The N2 subtype shows protumoral effects, while the N1 subtype shows antitumoral effects. TANs exhibit a more pronounced N1 phenotype in the presence of IFN-*β* around the tumor (28). In our case, considering that INI1 loss is associated with an IFN-rich environment, it can be concluded that the neutrophils were in the N1 phenotype, which has antitumoral effects.

### Literature review

Misdiagnosis of PDC as epithelioid sarcoma is exceedingly rare. In a literature search performed in PubMed/MEDLINE using the keywords *(“poorly differentiated chordoma”) AND (“epithelioid sarcoma”)*, and limited to English-language publications from database inception through January 27, 2026, we identified only a single report describing PDC initially misdiagnosed as epithelioid sarcoma. In the case reported by O’Connor et al., a 60-year-old man presenting with longstanding knee pain, initially attributed to osteoarthritis, underwent synovial histologic evaluation that demonstrated epithelioid-to-histiocytoid cells with high-grade nuclear atypia (18). The lesion was first considered metastatic carcinoma; however, based on morphologic features and SMARCB1/INI-1 loss, a diagnosis of epithelioid sarcoma was rendered. Upon subsequent re-review, strong nuclear brachyury positivity was demonstrated, and the diagnosis was revised to extra-axial poorly differentiated chordoma. Beyond the epithelioid sarcoma pitfall, misclassification of PDC as metastatic carcinoma has also been documented (29–31). Additionally, atypical teratoid/rhabdoid tumors (AT/RT) should also be considered in the differential diagnosis of PDC (32). In the pediatric setting, the differential diagnosis with atypical teratoid/rhabdoid tumor (AT/RT) is also challenging given the shared SMARCB1 loss; retrospective application of brachyury immunohistochemistry has led to diagnostic revisions as PDC in multiple reported series (33, 34).

## Conclusions

Extended immunohistochemistry can lead to reclassification of axial or paraspinal tumors initially interpreted as epithelioid sarcoma toward poorly differentiated chordoma, which may carry important implications for patient counseling, therapeutic decision-making, and surveillance planning. In INI-1–deficient epithelioid neoplasms arising in axial or paraspinal locations, poorly differentiated chordoma should be carefully considered in the differential diagnosis, and early incorporation of brachyury into the immunohistochemical workup may help reduce the risk of misclassification.

## Data Availability

The original contributions presented in the study are included in the article/Supplementary Material, further inquiries can be directed to the corresponding author.
